# Sponsorship of Internal Medicine Subspecialty Fellowships Since 2000: Trends and Community Hospital Involvement

**DOI:** 10.3885/meo.2009.Res00307

**Published:** 2009-07-14

**Authors:** Robert Ferguson, Fernanda Porto Carreiro, Lyn Camire

**Affiliations:** Department of Medicine, Union Memorial Hospital, Baltimore, MD, USA

**Keywords:** Specialists, workforce, supply

## Abstract

**Background:**

Since 2002, market studies have predicted a physician shortage with an increasing need for future subspecialists. A Residency Review Committee (RRC) rule that restricted sponsorship of fellowships was eliminated in 2005, but the influence of this change on the number of fellowships is not known. We believed that the rules change might make it possible for community hospitals to offer fellowships. Our objectives were to determine the extent of change in the number of fellowships in university and community hospitals from 2000 through 2008, both before and after the RRC regulation change in 2005, and to determine whether community hospitals contributed substantially to the number of new fellowships available to internal medicine graduates.

**Methods:**

We used archived Accreditation Council for Graduate Medical Education (ACGME) data from July 2000 through June 2008. The community hospital category included multispecialty clinics, community programs, and municipal hospitals.

**Results:**

Of the 94 newly approved internal medicine subspecialty fellowships in this time period, 59 (63%) were community sponsored. As of 6/02/08, all were in good standing. Thirteen programs were started as a department of medicine solo fellowship since 2005. The number of new programs approved between 2005 and 2008 was roughly three times the number approved between 2000 and 2004.

**Conclusions:**

The number of subspecialty fellowship programs and approved positions has increased dramatically in the last 8 years. Many of the new programs were at community hospitals. The change in RRC rules has been associated with increased availability of fellowship programs in the university and community hospital setting for subspecialty training.

Since 2002, market studies have predicted a physician shortage with an increasing need for future subspecialties. The Bureau of Health Professions report on physician supply and demand projects that, by the year 2020, the growth and aging of the United States population will increase the demand for physicians by 22 percent, with substantial increases in demand for subspecialists.^1^ It is in the interests of physicians in training to have access to subspecialty fellowship programs in both the university and community hospital setting.

Recommendations for increasing subspecialty training include increasing the number of fellowship programs, reducing the time needed to complete the training, and offering incentives to physicians entering a particular subspecialty.^2^ Increasing fellowship positions can result from either expanding existing programs or developing new ones. Subspecialty societies provide the major impetus for initiating new fellowships or expanding existing ones, but the number of fellowship programs has not been adequate in some areas to meet anticipated need.^3^ In 2005, the Internal Medicine Residency Review Committee (RRC) special requirements for Internal Medicine fellowships eliminated the requirement that a sponsoring institution sponsor at least three subspecialty fellowships. This requirement had potentially restricted the introduction of new fellowships in community hospitals because of the administrative and educational demands of introducing three fellowships at one time. Introduction of a single or solo fellowship program rather than three fellowships would be more manageable for community hospitals in particular.

We believed the 2005 change in RRC requirements would lead to an increase in the number of fellowships and institutional sponsors. We also believed community hospitals would sponsor additional fellowships as a result of this rule change. Our objectives were to determine the extent of change in the number of fellowships in university and community hospitals from 2000 through 2008, both before and after the RRC regulation change in 2005, and to determine whether community hospitals contributed substantially to the number of new fellowships.

## Methods

The Accreditation Council for Graduate Medical Education (ACGME) Internal Medicine RRC lists all of the Internal Medicine fellowships sponsored in the U.S.^4^ The ACGME database provides the general public with basic information about all ACGME-accredited programs and their sponsoring institutions, including the number of resident positions approved and filled and the present and past accreditation status of each program. We used this database to examine Internal Medicine fellowship programs between July 2000 and June 2008. At the time this study was done, the ACGME had not updated the 2008 data with the number of resident positions approved and the number of programs that have been withdrawn. Data in these two categories were reviewed through 2007. We last accessed the ACGME database on August 18, 2008.

The data were extracted from reports listing newly accredited fellowship programs by year. We distinguished community programs from non-community or university programs. We classified as university programs all programs listed as such by the Association of Program Directors in Internal Medicine, which included one sponsoring hospital for each medical school. In addition, we considered any federally sponsored program as a university program because of the similarities to university programs and lack of features common to community programs. For example, military and Veterans Administration programs’ organization, priorities, and relationships with medical schools are similar to university programs and unlike community programs. Thus, universities in our classification included traditional universities, military, and Veterans Administration-sponsored institutions. All other programs, including community programs, multispecialty clinics, and municipal programs, were considered community programs.

We looked at the major fellowships in hematology, oncology, hematology/oncology, pulmonary, critical care, pulmonary/critical care, endocrinology and metabolism, gastroenterology, cardiovascular disease, nephrology, rheumatology, geriatrics, and infectious disease. We did not include sub-subspecialty fellowships, such as cardiology angioplasty, in our analysis. We also looked at the current status of all new programs introduced between July 2000 and June 2008 to determine if any were not in good standing.^4^ We did not look at actual fellows because that is influenced by other factors besides RRC approval, such as institutional funding. Staffing of fellowships is an institution's prerogative, whereas the RRC deals purely with accreditation.

We used the number of programs and fellowship positions accredited as of July 2000 as a baseline for analyzing the trends in fellowship training between July 2000 and June 2008.

## Results

**Figure 1.**
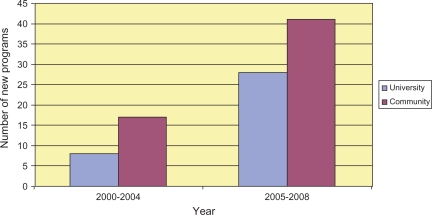
Number of university versus community internal medicine programs created before and after 2005 RRC regulation change

Ninety-four new subspecialty programs were accredited between July 2000 and 2008 as of the January 2008 meeting of the Internal Medicine RRC (Table 1). Cardiovascular disease, geriatric medicine, nephrology, and pulmonary/critical care had the most new programs. Of the 94 new programs, 59 were in community programs (63%). Of 13 new cardiology programs, 12 were in community hospitals. Community programs also had a majority in infectious diseases (5/6), nephrology (8/12), and pulmonary disease/critical care (8/12). Of the 94 new programs, 25 were approved between July 2000 and June 2004 and 69 between July 2004 and June 2008 (Figure 1).

**Table 1.** New internal medicine fellowship programs July 2000 through June 2008

	No. (%) new programs		
		
Subspecialty	Community	University	New programs (% of total)^*^
Cardiovascular disease	12 (92)	1 (8)	13 (14)
Critical care medicine	3 (75)	1 (25)	4 (4)
Endocrinology	3 (33)	6 (67)	9 (10)
Gastroenterology	5 (83)	1 (17)	6 (6)
Geriatric medicine	7 (58)	5 (42)	12 (13)
Hematology	0	0	0 (0)
Hematology and oncology	5 (56)	4 (44)	9 (10)
Infectious disease	5 (83)	1 (17)	6 (6)
Nephrology	8 (67)	4 (33)	12 (13)
Oncology	0	0	0 (0)
Pulmonary disease	1 (33)	2 (67)	3 (3)
Pulmonary disease and critical care	8 (67)	4 (33)	12 (13)
Rheumatology	2 (25)	6 (75)	8 (9)
All programs	59 (63)	35 (37)	94 (100)

^*^Percentages do not total 100% because of rounding.

New York, with 19 new fellowships, was heavily represented in terms of new programs. Florida had 11 new fellowships, followed by California with 6 and Ohio, Michigan, and Texas with 5 each. Prior to the 2005 regulation change, there were four solo fellowships, all in community programs. Between July 2004 and June 2008, 13 institutions became sponsors of solo fellowships, with 12 of the 13 in community programs.

All 94 new programs are in good standing according to the current RRC website.^4^ During the 2000 to 2007 interval, 102 existing programs were withdrawn. A number of fellowships in oncology, hematology, pulmonology, and critical care were withdrawn and included in combined programs. Of the 102 withdrawn programs, 20 had lost RRC accreditation and 82 were voluntarily withdrawn.

Community hospitals sponsored 28% of the total subspecialty fellowships in 2007(Table 2). The greatest increase in subspecialty fellowship numbers between 2000 and 2007 was in hematology/oncology with 602 newly approved positions, an increase of 83% over the 7-year period (Table 3). This increase has coincided with a decline in oncology positions without a hematology component, which were reduced by 51 during this time period, and hematology fellowships, the number of which fell by 50. The increase in pulmonary disease and critical care was similarly associated with a decrease in numbers of separate pulmonary and critical care fellows.

**Table 2.** Number of community and university hospital programs by subspecialty in June 2007

	No. (%) programs		
		
Subspecialty	Community	University	New programs (% of total)
Cardiovascular disease	67 (38)	110 (62)	177 (14)
Critical care medicine	7 (23)	23 (77)	30 (2)
Endocrinology	20 (16)	102 (84)	122 (10)
Gastroenterology	49 (32)	106 (68)	155 (12)
Geriatric medicine	32 (31)	70 (69)	102 (8)
Hematology	2 (22)	7 (78)	9 (1)
Hematology and oncology	37 (29)	90 (71)	127 (10)
Infectious disease	32 (23)	110 (77)	142 (11)
Nephrology	41 (29)	98 (71)	139 (11)
Oncology	7 (44)	9 (56)	16 (1)
Pulmonary disease	18 (69)	8 (31)	26 (2)
Pulmonary disease and critical care	29 (22)	101 (78)	130 (10)
Rheumatology	14 (13)	95 (87)	109 (8)
Total	355 (28)	929 (72)	1284 (100)

**Table 3.** Change in number of positions by subspecialty July 2000 through June 2007

	No. positions		
		
Subspecialty	2000	2007	Change in no. positions (%)
Cardiovascular disease	2118	2328	210 (10)
Critical care medicine	156	165	9 (6)
Endocrinology	372	519	147 (40)
Gastroenterology	910	1283	373 (41)
Geriatric medicine	291	318	27 (9)
Hematology	96	46	−50 (−52)
Hematology and oncology	724	1326	602 (83)
Infectious disease	594	701	107 (18)
Nephrology	628	845	217 (35)
Oncology	170	119	−51 (−30)
Pulmonary disease	142	82	−60 (−42)
Pulmonary disease and critical care	857	1237	380 (44)
Rheumatology	287	394	107 (37)

## Discussion

Since 2000, the prediction of staffing shortages in medical subspecialties have led many subspecialty organizations to call for an increase in fellowship positions. The American College of Cardiology cited a growing shortage of cardiologists that would impair patient access to care and hinder cardiovascular research programs as academic cardiologists devote more time to patients.^2^ In nephrology, the number of ACGME-approved fellowship programs is increasing, but not fast enough to produce the extra 200 trainees per year projected as necessary to meet 2010 workforce demands.^3^ Oncology is experiencing physician shortages as a result of the aging population and the increasing number of cancer survivors. The American Society of Clinical Oncology Workforce Study projects a shortage of 3800 oncologists by 2020.^5^ Critical care, endocrinology, and rheumatology are also unprepared for the aging population, citing current shortages and increasing demands for subspecialists in the coming decade.^6^–^9^ New technology, more complex patient care, and professional burnout is blamed for an increase in demand for subspecialists in infectious disease.^10^ Although the argument for increasing subspecialty training is not uniformly accepted,^11^, it appears increased subspecialty training is considered a high priority.

No consensus has been reached regarding the best solution to the projected physician subspecialist workforce shortage, but many groups are proposing recommendations to increase the number of subspecialists.^2^ Increasing fellowship positions can result from either expansion of existing programs or development of new ones. The elimination in 2005 of an RRC rule requiring sponsorship of at least three fellowships removed a restriction that put fellowship sponsorship out of reach, especially for community hospitals. Our findings suggest that this rule change has encouraged a substantial increase in available subspecialty fellowships, including many new programs in community hospitals.

Besides the benefit of meeting anticipated workforce needs, additional fellowships meet the requirements of internal medicine graduates who are increasingly demanding the option to subspecialize. The lack of subspecialty training after residency may adversely affect a hospital's residency programs. The change in rules has allowed the rapid expansion of solo fellowships, which is a benefit to the hospitals sponsoring them and also to the graduates filling the positions.

Our data show a dramatic increase in fellows and fellowship sponsorship in this last decade, particularly in the last four years since the RRC changed the special requirements. This impact has happened in places where there are many community programs, such as New York State, and the bulk of the new programs are in community hospitals, many of which are sponsoring fellowships for the first time. There has been an anecdotal perception that the RRC does not favor community hospital fellowships and that loss of accreditation after early accreditation visits would be an issue for various reasons, including not fulfilling research requirements. However, this does not appear to be the case. None of the recently accredited fellowships have been identified with problems as of 2008.

This study confirms that the change in rules in 2005 has allowed a substantial expansion of subspecialty fellowships available to internal medicine graduates. Community hospitals have increasingly contributed to this expansion in programs as a result of the change in rules, adding to the subspecialty training choices available to internal medicine graduates.
